# *Parvimonas micra* as a putative non-invasive faecal biomarker for colorectal cancer

**DOI:** 10.1038/s41598-020-72132-1

**Published:** 2020-09-17

**Authors:** Thyra Löwenmark, Anna Löfgren-Burström, Carl Zingmark, Vincy Eklöf, Michael Dahlberg, Sun Nyunt Wai, Pär Larsson, Ingrid Ljuslinder, Sofia Edin, Richard Palmqvist

**Affiliations:** 1 Department of Medical Biosciences, Pathology, Umeå University, Building 6M, 90185 Umeå, Sweden; 2 Department of Surgery, Sunderby Hospital, Luleå, Sweden; 3 Department of Molecular Biology, Umeå University, Umeå, Sweden; 4 Department of Radiation Sciences, Oncology, Umeå University, Umeå, Sweden

**Keywords:** Cancer, Biomarkers, Gastroenterology

## Abstract

The use of faecal microbial markers as non-invasive biomarkers for colorectal cancer (CRC) has been suggested, but not fully elucidated. Here, we have evaluated the importance of *Parvimonas micra* as a potential non-invasive faecal biomarker in CRC and its relation to other microbial biomarkers. The levels of *P. micra*, *F. nucleatum* and *clbA* + bacteria were quantified using qPCR in faecal samples from a population-based cohort of patients undergoing colonoscopy due to symptoms from the large bowel. The study included 38 CRC patients, 128 patients with dysplasia and 63 controls. The results were validated in a second consecutive CRC cohort including faecal samples from 238 CRC patients and 94 controls. We found significantly higher levels of *P. micra* in faecal samples from CRC patients compared to controls. A test for *P. micra* could detect CRC with a specificity of 87.3% and a sensitivity of 60.5%. In addition, we found that combining *P. micra* with other microbial markers, could further enhance test sensitivity. Our findings support the potential use of *P. micra* as a non-invasive biomarker for CRC. Together with other microbial faecal markers, *P. micra* may identify patients with “high risk” microbial patterns, indicating increased risk and incidence of cancer.

## Introduction

The prognosis in colorectal cancer (CRC) depends to a large extent on tumour stage at diagnosis and advanced disease is associated with poorer life expectancy. Current screening strategies are primarily based on detection of human faecal haemoglobin (F-Hb) and colonoscopy, with the latter being both time consuming, costly and uncomfortable for the patient. Recent studies have highlighted the importance of the gut microbiota in CRC development^1–5^, and a better understanding of the microbial patterns connected to CRC may serve to improve the accuracy and accessibility of today’s CRC screening.

Accumulating evidence suggests that dysbiosis of the gut increases the risk of developing CRC^6–11^. Studies have shown a structural segregation of bacterial species between CRC patients and healthy subjects. Some bacteria are altered or more frequently present in patients with CRC, whereas some species are suppressed compared to healthy individuals^12^. Lately, a microbial driver-passenger model for carcinogenesis has been suggested, where some bacteria promote cancer while others accumulate due to higher fitness in the resulting altered microenvironment^13^. Bacteria associated with carcinogenesis (drivers) often produce genotoxins that cause double stranded DNA breakage and mutations that can lead to tumour initiation and progression. Bacteria associated with inflammatory responses accelerating tumour progression can be drivers, but are more often thought to be passengers^14^.

*Fusobacterium nucleatum* is a part of the commensal gut and oral cavity flora, but has been linked to pathological conditions including appendicitis, inflammatory bowel disease (IBD), periodontitis and CRC^15–18^. Preclinical models have demonstrated multiple mechanisms by which *F. nucleatum* may promote CRC progression, including E-cadherin-mediated activation of Wnt/β-catenin signalling^15^. *F. nucleatum* has also been suggested to negatively regulate the anti-tumor immune response^19^. Colibactin toxin-producing (c*lbA* +) bacteria have been suggested to promote CRC development through inducing double-stranded DNA breaks and cellular senescence^20^. However, recently the role of colibactin as a potential driver has been debated^21^. *Parvimonas micra* is, like *F. nucleatum,* commensal in the oral cavity and has been linked to pathogenesis leading to intracranial abscesses, pericarditis and necrotising fasciitis, as well as CRC^4,14,22–25^. However, the role of *P. micra* in CRC progression is still largely unknown, and the potential of *P. micra* as a faecal marker for CRC detection has not been fully elucidated.

Using faecal microbiota in CRC screening would serve as a non-invasive complement to today’s F-Hb screening and could further identify patients that would benefit from colonoscopy. Our group has earlier published a study showing associations between *F. nucleatum* and *clbA* + bacteria and CRC^2^. In the present study, we investigated the potential of *P. micra* as a non-invasive faecal marker for detection of CRC using the same cohort. Our findings were further validated in a second larger cohort. The diagnostic performance of combined tests of *P. micra*, *F. nucleatum* and *clbA* + bacteria were also evaluated.

## Results

### *P. micra* is more abundant in faeces of patients with CRC

The level of *P. micra* was analysed in faecal samples from 38 cancer patients, 128 patients with dysplasia, and 63 matched controls from the FECSU cohort, which is a population-based cohort of patients undergoing colonoscopy due to large bowel symptoms. Our findings were further validated using faecal samples from 238 CRC patients and 94 matched controls from the U-CAN cohort of consecutive CRC patients. The clinical characteristics of the study patients from the FECSU and U-CAN cohorts can be found in Table 1 and Table 2, respectively.

**Table 1:** Clinical characteristics of study patients from the FECSU cohort.

	Total	Control	Dysplasia	Cancer
	n = 229	n = 63	n = 128	n = 38
**Age (%)**				
≤ 59	33 (14.4)	6 (9.5)	23 (18.0)	4 (10.5)
60–69	88 (38.4)	21 (33.3)	55 (43.0)	12 (31.6)
70–79	82 (35.8)	26 (41.3)	39 (30.5)	17 (44.7)
≥ 80	26 (11.4)	10 (15.9)	11 (8.6)	5 (13.2)
**Gender (%)**				
Female	103 (45.0)	30 (47.6)	55 (43.0)	18 (47.4)
Male	126 (55.0)	33 (52.4)	73 (57.0)	20 (52.6)
**Location (%)**	n = 166			
Right colon	45 (27.1)	n.a	34 (26.6)	11 (28.9)
Left colon	74 (44.6)	n.a	57 (44.5)	17 (44.7)
Rectum	47 (28.3)	n.a	37 (28.9)	10 (26.3)
**Stage (%)**				
**I**		n.a	n.a	2 (5.4)
II		n.a	n.a	20 (54.1)
III		n.a	n.a	8 (21.6)
IV		n.a	n.a	7 (18.9)

*Abbreviations:* n.a., not applicable.

**Table 2:** Clinical characteristics of study patients from the U-CAN cohort.

	Total	Control	Cancer
	n = 332	n = 94	n = 238
**Age (%)**			
≤ 59	56 (16.9)	15 (16.0)	41 (17.2)
60–69	120 (36.1)	33 (35.1)	87 (36.6)
70–79	113 (34.0)	33 (35.1)	80 (33.6)
≥ 80	43 (13.0)	13 (13.8)	30 (12.6)
**Gender (%)**			
Female	136 (41.0)	41 (43.6)	95 (39.9)
Male	196 (59.0)	53 (56.4)	143 (60.1)
**Location (%)**			
Right colon		n.a	48 (20.2)
Left colon		n.a	41 (17.2)
Rectum		n.a	149 (62.6)
**Stage (%)**			
I		n.a	46 (20.4)
II		n.a	77 (34.2)
III		n.a	65 (28.9)
IV		n.a	37 (16.4)

n.a., not applicable.

**Figure 1:**
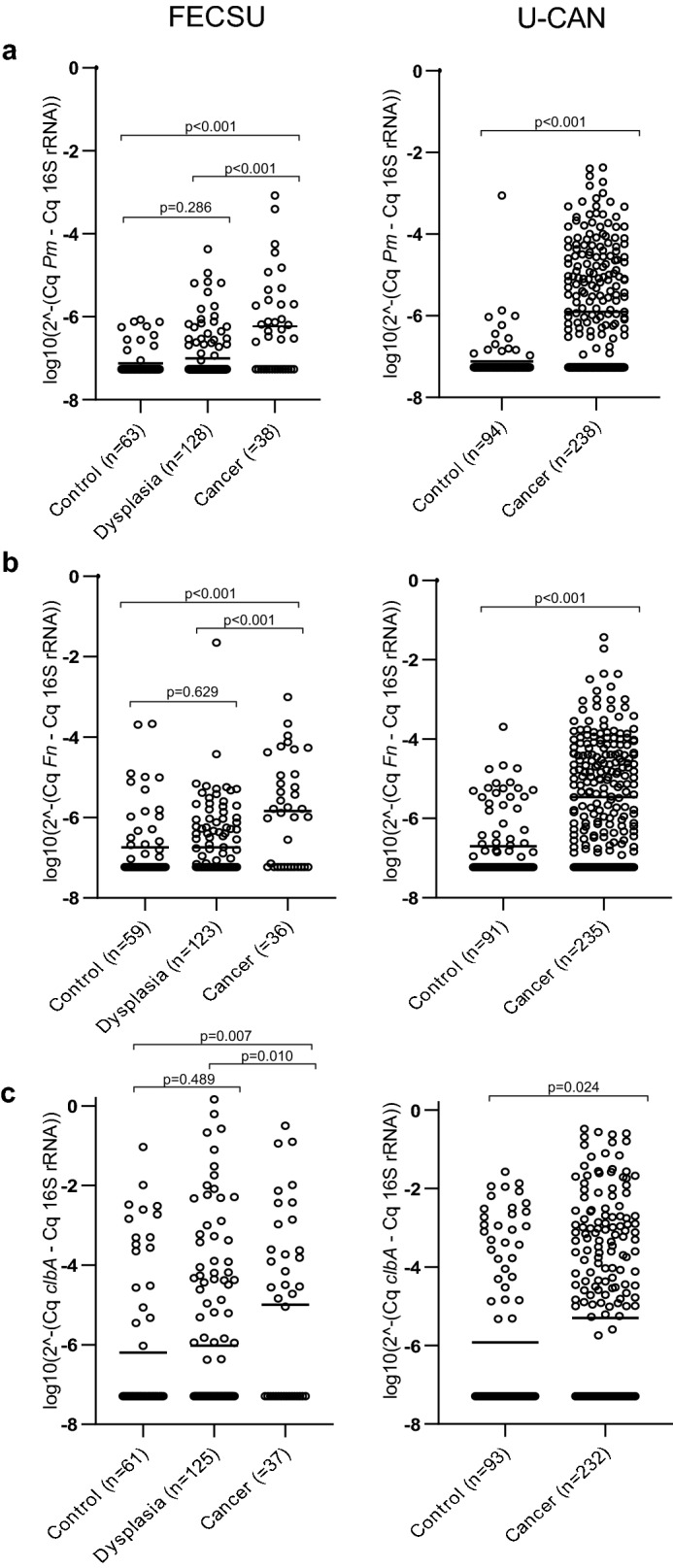
Increased levels of specific microbial markers are detected in faeces of CRC patients. Scatter plots are used to illustrate the relative levels of (**A**) *P. micra* (*Pm*), (**B**) *F. nucleatum* (*Fn*), and (**C**) *clbA* + bacteria (*clbA*) in faeces of control patients, and patients diagnosed with dysplasia or CRC from the FECSU and U-CAN cohorts. Horizontal lines indicate mean relative expression calculated by the 2^-ΔCq^ method with the total microbial 16S rRNA gene DNA as reference.

**Figure 2:**
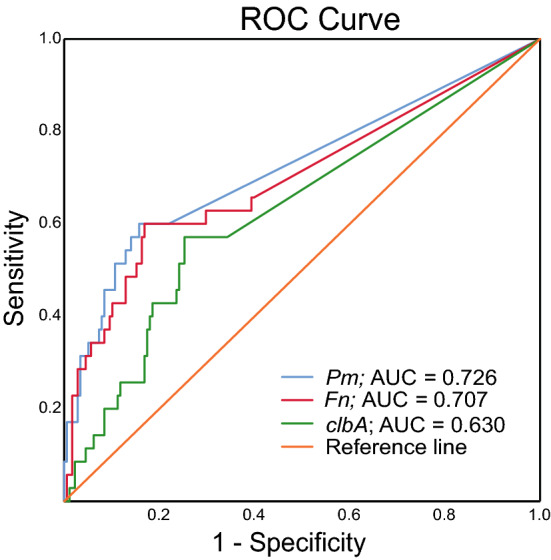
ROC curves displaying the specificity and the sensitivity for *P. micra* (*Pm*), *F. nucleatum* (*Fn*), and *clbA* + bacteria (*clbA*) to detect CRC. ROC-curves were calculated using the levels for the specific marker as indicated and cancer/no cancer. The levels of a specific marker in each sample was given as a relative quantification calculated by the 2^-ΔCt^ method with the total microbial 16S rRNA gene DNA as reference.

**Figure 3:**
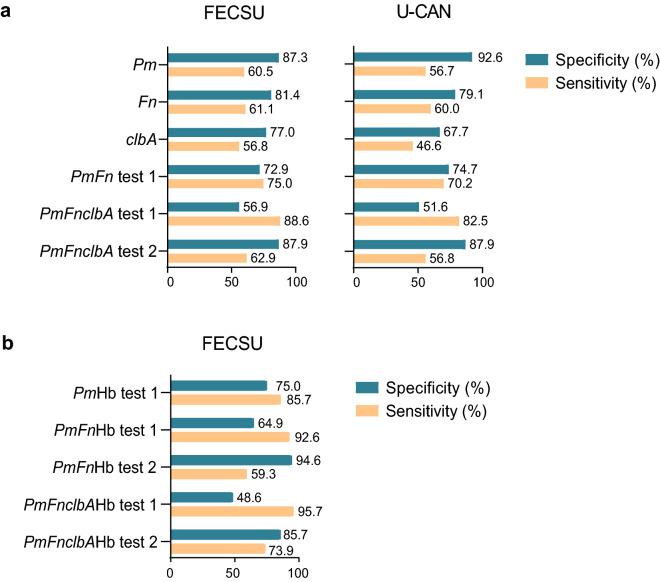
Performance of single faecal microbial markers or combinations of markers in CRC detection. Sensitivity and specificity for CRC detection is displayed for a test of (**A**) *P. micra* (*Pm*), *F. nucleatum* (*Fn*), or *clbA* + bacteria (*clbA*), as well as combined tests using several microbial markers for the FECSU and U-CAN cohort, and (**B**) for combined tests using microbial markers and immunochemical F-Hb (Hb) for the FECSU cohort. For test 1, a positive test result was given to samples with at least one positive marker. For test 2, a positive test result was given to samples with at least two positive markers.

A qPCR assay targeting the *rpoB* gene was used to detect *P. micra*. *P. micra* was significantly more abundant in faecal samples from CRC patients compared to controls in the FECSU cohort (*P* < 0.001; Fig. 1A). No significant difference was found between patients with dysplasia and controls (*P* = 0.286), and significantly higher levels of *P. micra* were found in samples from CRC patients compared to patients with dysplasia (*P* < 0.001). The finding of significantly higher levels of *P. micra* in faecal samples from CRC patients compared to controls could be validated in the U-CAN cohort (*P* < 0.001; Fig. 1A). The area under the ROC curve for detection of CRC for the FECSU cohort was 0.726 (Fig. 2). A cut-off (2.5 × 10^-7) for a positive detection of *P. micra* was selected using Youden’s index. Using the optimised cut-off, a test for *P. micra* could detect cancer with a sensitivity of 60.5% and a specificity of 87.3% in the FECSU cohort (Table 3; Fig. 3A). For the U-CAN cohort, the sensitivity was 56.7% and the specificity was 92.6% (Fig. 3A). No clear associations of *P. micra* with clinical characteristics of CRC patients of the FECSU and U-CAN cohorts were found, including tumour stage and site (Supplementary Table S1 and S2).

**Table 3:** Microbial alterations in faeces of study patients from the FECSU cohort.

	Total	Control	Dysplasia	Cancer	*P*-value
	n = 229	n = 63	n = 128	n = 38	
***P. micra (%)***					
Low	177 (77.3)	55 (87.3)	107 (83.6)	15 (39.5)	< 0.001
High	52 (22.7)	8 (12.7)	21 (16.4)	23 (60.5)	
***F. nucleatum (%)***					
Low	166 (76.1)	48 (81.4)	104 (84.6)	14 (38.9)	< 0.001
High	52 (23.9)	11 (18.6)	19 (15.4)	22 (61.1)	
***clbA + bacteria (%)***					
Low	154 (69.1)	47 (77.0)	91 (72.8)	16 (43.2)	0.001
High	69 (30.9)	14 (23.0)	34 (27.2)	21 (56.8)	

*χ*^2^ tests were used to compare categorical variables.

### The relative abundance of *P. micra*, *F. nucleatum* and *clbA* + bacteria in CRC patients

*F. nucleatum* and *clbA* + bacteria have been previously assessed in the FECSU cohort^2^. Here, DNA was extracted from another aliquot of the same stool sample using an improved technique as described below. Furthermore, in this study we used other established PCR assays from the literature to detect both *F. nucleatum* and the 16S rRNA gene. While the *clbA* assay is the same as in the previous study, we here present a relative quantification using the 16S rRNA gene. Previous findings of significantly enriched levels of *F. nucleatum* and *clbA* + bacteria in faeces of CRC patients could be replicated in the current study and validated in the U-CAN cohort (Fig. 1B, C). The area under the ROC-curve was for this study 0.707 for *F. nucleatum* and 0.630 for *clbA* + bacteria (Fig. 2). Using the cut-offs of 9.5 × 10^-7 for *F. nucleatum* and 8.8 × 10^-6 for *clbA* + bacteria, *F. nucleatum* could detect CRC with a sensitivity of 61.1% and a specificity of 81.4%, and *clbA* + bacteria detected CRC with a sensitivity of 56.8% and a specificity of 77.0% in the FECSU cohort (Table 3; Fig. 3A). These results were further validated in the U-CAN cohort, with *F. nucleatum* showing a sensitivity of 60.0% and specificity of 79.1% to detect CRC, and *clbA* + bacteria showing a sensitivity of 46.6% and specificity of 67.7% (Fig. 3A).

**Figure 4:**
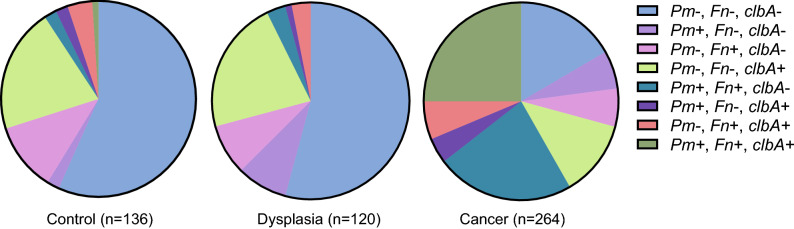
The distribution of specific microbial markers in faeces of CRC patients. Circle diagrams are used to illustrate the abundance of *P. micra* (*Pm*), *F. nucleatum* (*Fn*) and *clbA* + bacteria (*clbA*) in faecal samples with all three markers evaluated of control patients, and patients diagnosed with dysplasia or CRC from the FECSU and U-CAN cohorts.

When dissecting the relative abundance of *P. micra*, *F. nucleatum* and *clbA* + bacteria in faecal samples, we found a quite similar pattern between controls and dysplasias, and a severely altered pattern in CRC patients (Fig. 4). *P. micra* was rarely detected as the sole bacterial marker in faecal samples from CRC patients, but was instead often found in combination with *F. nucleatum (r*_*s*_ 0.620; *P* < 0.001), with or without *clbA* + bacteria. Faecal samples in which *P. micra* or *F. nucleatum* were found in combination with *clbA* + bacteria alone were rare. These findings may indicate a cooperative effect between some bacterial markers, *P. micra* and *F.* nucleatum in particular, in the carcinogenic process.

### A combined test of faecal *P. micra*, *F. nucleatum* and *clbA* + bacteria predicts CRC

We further explored the performance of combined tests of *P. micra* with other microbial markers. A combined test of *P. micra* with *F. nucleatum,* or *F. nucleatum* and *clbA* + bacteria, where a positive test result was described as one or more positive marker, enhanced sensitivity to 75.0%, or 88.6%, respectively, but with the corresponding decrease in specificity to 72.9% and 56.9%, respectively (Fig. 3A). Similar results were found also for the U-CAN cohort, with a sensitivity of 70.2%, and 82.5%, and a specificity of 74.7% and 51.6%, respectively for *P. micra* in combination with *F. nucleatum* alone or together with *clbA* + bacteria (Fig. 3A). A more restricted test, where a positive test result was given to faecal samples positive for two or more markers, did not improve the quality of detection compared to *P. micra* alone (Fig. 3A).

### The performance of *P. micra* in combined tests with microbial markers and/or F-Hb

We previously reported the diagnostic performance of immunochemical F-Hb for CRC detection in the FECSU cohort^2^. Here, we further analysed if the performance for a test of *P. micra* alone or with additional microbial markers could be enhanced by the addition of F-Hb. Since F-Hb was only available for the FECSU cohort, these studies were restricted to this cohort. We found that addition of F-Hb could enhance the sensitivity of the test for *P. micra* to 85.7%, while decreasing the specificity to 75.0% (Fig. 3B). Addition of F-Hb to the test of *P. micra* and *F. nucleatum*, as well as *F. nucleatum* and *clbA* + bacteria, further enhanced the sensitivity to 92.6% and 95.7%, respectively (Fig. 3B). However, the addition of F-Hb reduced specificity of these tests to 64.9% and 48.6%, respectively (Fig. 3B). A more restricted test, where a positive test result was given to faecal samples with two or more positive markers, restored specificity to 94.6% and 85.7%, respectively, but with the consequence of reduced sensitivity to 59.3% and 73.9%, respectively, for the combination of F-Hb with *P. micra* in combination with *F. nucleatum* alone or together with *clbA* + bacteria (Fig. 3B).

## Discussion

In this study, we used targeted qPCR assays to investigate faecal microbial markers for CRC detection and applied a second larger cohort for validation. To apply qPCR for detection is an affordable and clinically very relevant approach. We found a significantly higher abundance of *P. micra* in faecal samples from CRC patients compared to controls. A test for *P. micra* in faeces could detect cancer with a sensitivity of 60.5% or 56.7% and a specificity of 87.3% or 92.6% in the FECSU and U-CAN cohorts, respectively. We further showed that the sensitivity of the assay could be enhanced by adding microbial markers *F. nucleatum* and *clbA* + bacteria, as well as F-Hb, but with a resulting decrease in specificity. This study thereby suggests *P. micra* as a candidate microbial marker for a non-invasive screening panel, with the potential of improving the diagnostic performance.

Our findings are supported by previous studies showing an alteration of *P. micra* in both faeces and tissue samples from CRC patients compared to controls. Findings of *P. micra* in faeces were mainly based on 16S rRNA gene amplicon sequencing^4,22–26^, but a few studies have also applied targeted qPCR assays^25,27^. In a study by Yu et al., a microbial signature was identified, including *P.micra*, which could distinguish CRC metagenomes from controls in several independent cross-ethnic cohorts^25^. They further employed qPCR measurements of *P. micra* and *F. nucleatum*, and demonstrated that combined analyses of these two markers could accurately classify patients with CRC. In the present study, we proceed from a population-based cohort better representing a true screening cohort. This cohort, in addition to CRC patients, also included patients with dysplasia. However, no significant difference in levels of *P. micra* between faecal samples from controls and dysplasias could be found. This suggests that *P. micra* is not present in pre-cancerous lesions and is more likely a passenger rather than a driver of tumourigenesis, a conclusion also supported by findings of Wong et al*.*^27^ Our results therefore indicate that *P. micra* represents as a poor detection marker of pre-cancerous lesions, which is not optimal from a screening perspective. *P. micra* is however found evenly distributed throughout all stages of CRC, suggesting that early stages of CRC can be identified, which was also suggested in the study by Yu et al.^25^.

In this study, *P. micra* was found to detect CRC with a similar sensitivity as *F. nucleatum*. However, fewer control samples presented with a positive test result for *P. micra*, resulting in a higher specificity of testing. Interestingly, *P. micra* was found to be highly correlated to *F. nucleatum* in faeces*,* and similar findings were presented by Yu et al*.*^25^ These bacteria, both being oral pathogens, may therefore interact in the carcinogenic process. The pathogenicity of *F. nucleatum* in CRC has been suggested to be mediated partly through stimulation of inflammatory processes^28^. Little is known about the role of *P. micra* in CRC progression, but it may be that *P. micra* and *F. nucleatum* interact to potentiate a pro-inflammatory microenvironment. Interestingly, *P. micra* and *F. nucleatum* have been shown to aggregate and form biofilms in vitro*.*^29^ Additionally, one study on periodontitis indicated that *P. micra* may stimulate immunity through interactions with pattern-recognition NOD2 receptors^30^. Further studies are needed to elucidate a possible pro-tumourigenic role of *P. micra*.

The population-based cohort used in this study included patients who had undergone a colonoscopy at the University Hospital in Umeå, Sweden. Indications for colonoscopy were gastrointestinal symptoms of large bowel disease, visible blood in faeces and/or positive F-Hb. Thus, even though regarded as healthy, patients in the control group still manifested with bowel symptoms that could be linked to an altered gut microbiota. Therefore, it is possible that the specificity of a combined test of microbial markers would be improved in a randomized screening cohort. There is considerable inter-individual variation of the gut flora, and many factors including lifestyle, age, genetics and medication, especially antibiotics treatment, affect the composition. In order to avoid this type of possible bias, we designed control groups matched in age and gender. Also, no patients included had ongoing antibiotic treatment, even though a previous antibiotic treatment could still have altered the gut microbiota. Furthermore, our study included a validation cohort, which increases the chance for true positive results. The microbial composition is also affected by the faecal sampling and sample storage. In this study, faeces from a single randomly taken sample was used. Even though appealing from a clinical perspective, using a single sample from a small amount of stool increases the risk of a non-representative result. In order to achieve more reliable results, repetitive faecal samples would be preferred. It should be noted also that this study was not based on a randomized screening cohort, and the efficacy of a test quantifying *P. micra* in CRC screening therefore remains to be evaluated.

Using faecal samples for screening provides an easy non-invasive method. If used in the clinic, screening participation would likely increase and thereby also the potential for early detection and patient survival. Today, F-Hb is the most used non-invasive screening method. Tests for immunochemical F-Hb (FIT) are however poor at detecting non-bleeding lesions and do not have high enough sensitivity to detect advanced adenoma or cancer^31^. In this study, a test for *P.micra* alone had a slightly decreased sensitivity compared to immunochemical F-Hb to detect CRC^2^. A combined test of *P.micra* and F-Hb had superior sensitivity compared to F-Hb alone, which could further be enhanced by additional microbial markers. Combined analyses of *P. micra* and F-Hb in faecal samples from CRC patients has been assessed by Wong et al., showing similar results^27^. Microbial markers could thereby serve as a complement to F-Hb screening in order to find non-bleeding lesions, to increase the sensitivity of the test, and to better specify patients for further colonoscopy examination. Finding risk patterns using a larger number of microbial markers is likely to increase the accuracy of the method. If a patient would be identified by a high-risk microbial pattern, a colonoscopy would however still be needed in order to verify the diagnosis. Patients without colonoscopy findings, would need to be followed regularly by repetitive colonoscopy exams, which may lead to an unnecessary psychological burden.

In conclusion, *P. micra* is a promising candidate for a future faecal non-invasive combined CRC screening test including microbial markers and F-Hb. We suggest that detection of “high risk” microbial patterns may facilitate the finding of patients with increased CRC cancer risk. Future studies combining different microbial markers could possibly enhance the tests accuracy. These studies may also lead to a better understanding of the role of bacteria in CRC tumour development and progression.

## Materials and methods

### Study cohort

This study is based on cohorts from the Faecal and Endoscopic Colorectal Study in Umeå (FECSU) and the Uppsala-Umeå Comprehensive Cancer Consortium (U-CAN). The FECSU cohort includes patients who underwent colonoscopy at the University Hospital in Umeå, Sweden, between the years 2008–2013, and has been previously described^2^. In brief, indications for colonoscopy were gastrointestinal symptoms that may indicate large bowel disease, visible blood in faeces and/or positive F-Hb. Exclusion criteria were colonoscopy within one week, dementia and low performance status, including mental and physical disabilities. All colonoscopies were performed according to standard routines at the endoscopy unit. Biopsies were taken when clinically relevant and evaluated by a pathologist in clinical routine handling. All neoplastic lesions were further subdivided into low grade dysplasia, high grade dysplasia and adenocarcinoma. In cases where multiple lesions were found, the most severe was used for classification. In total, 1997 patients were invited to participate in the FECSU study. 861 patients denied participation, leaving 1,136 patients included. Of these, 39 were diagnosed with CRC and 135 with low or high grade dysplasia. The U-CAN project is a collaboration between Umeå University and Uppsala University, which longitudinally collects blood, tissue, faeces, radiological data, and clinical data over time from all enrolled CRC patients. In Umeå, more than 1,200 patients with CRC have been recruited since the start in 2010. Stool sample collection was performed during the years 2010 to 2014. During these years a total of 684 patients were included, out of which 260 CRC patients (38%) left a stool sample prior to the start of treatment.

Few patients (n = 14) were included in both cohorts, but separate stool samples were collected for the different studies and therefore these patients were not excluded.

The study protocol was approved by the Regional Ethical Review Board in Umeå, Sweden (dnr 08-184 M and dnr 2016/219–31), and in accordance with relevant guidelines and regulations. All included individuals have signed a written form of consent.

### Study patients included

For FECSU^2^, depletion of faecal samples from some patients resulted in inclusion of a total of 38 cancer patients, 128 patients with dysplasia and 63 controls for this study. Controls were selected from the patients recorded with no pathological findings and were matched by age and gender. Patients with IBD and hyperplastic polyps were excluded from the controls.

For U-CAN, limiting amounts of DNA extracted from the faecal samples resulted in a total of 238 patients included in the study. One hundred controls were density matched by age and gender and selected from the FECSU cohort, using the criteria described above. After exclusions due to depleted faecal samples or limited amounts of DNA extracted, 94 controls remained in the study.

### Stool sample collection and storage

Stool samples were collected by the patients in their home. Tubes for stool sample collection and study information were either sent by post together with the invitation for colonoscopy (for FECSU patients, as previously described^2^) or given to the patients at the time of diagnosis (U-CAN patients). For the FECSU cohort, included patients were asked to leave stool samples before starting the pre-colonoscopy cleansing procedure. Stool samples from CRC patients of the U-CAN cohort were collected before the start of cancer treatment. For both cohorts, stool tubes assigned for DNA extraction and microbial analyses contained 5 ml of preservative buffer, RNA*later* (Ambion), and were stored for a maximum of 7 days at room temperature prior to centrifugation for 20 min at 2000 rpm, disposal of excess fluid, and freezing at -80 °C. According to previous results, RNA*later* was shown to preserve both DNA yield and quality of stool samples stored at room temperature^2^.

### Detection of microbial markers in faeces using quantitative real-time PCR (qPCR)

DNA was extracted from approximately 0.2 g stool using the QIAamp PowerFecal DNA kit.

(Qiagen) according to the manufacturer’s instructions. In previous work of the FECSU cohort^2^, DNA was extracted using a different kit. Since the QIAamp PowerFecal DNA kit showed superior DNA yield and quality, DNA was re-extracted from another aliquot of the stool sample for the FECSU cohort. *P. micra*, *F. nucleatum* and *clbA* + bacteria were detected in the DNA by qPCR. All reactions were run in duplicates utilising the QuantStudio™ 6 Flex Real-Time PCR System (Applied Biosystems). In case of discrepancies in Cq values between duplicates (standard deviation > 0.5), the sample was rerun in duplicates 1–2 times until a stable duplicate was obtained. Samples with poor PCR performance consistently showing discrepant duplicates were excluded from the analysis. These exclusions included 11 and 6 faecal samples from FECSU and U-CAN cohorts, respectively, for the *F. nucleatum* assay, and 6 and 7 faecal samples from the FECSU and U-CAN cohorts, respectively, for the *clbA* assay. Primers and probes used for the different assays have been previously described and are listed with references in Supplementary Table S3. The performance of the qPCR assays was verified by analyses of replicates, serial dilutions, melting curves, and separation on agarose gels. qPCR efficiencies were stable and comparable between different amplicons and between SYBR Green I and TaqMan probe based assays. Markers not amplified within 38 cycles were defined as negative. *P. micra* 20,468 (DSZM), *F. nucleatum subsp. nucleatum Knorr* (ATCC 25,586), and *Escherichia coli* Nissle 1917 were used as positive controls for the respective PCR reactions. The levels of *P. micra, F. nucleatum, and clbA* + *bacteria* were presented as a relative quantification with the total microbial content using the 16S rRNA gene as reference as validated in the literature (Supplementary Table S3) and calculated using the 2^—^^ΔCt^ method. Cycle conditions used were as follows: For *P. micra* and *F. nucleatum* – 2 min at 50 °C, 10 min at 95 °C, followed by 40 cycles of: 95 °C for 15 s, 60 °C for 45 s: For *clbA* + bacteria—2 min at 50 °C, 10 min at 95 °C, followed by 40 cycles of: 95 °C for 15 s, 60 °C for 30 s, and 72 °C for 1 min: For 16S rRNA,—2 min at 50 °C, 10 min at 95 °C, followed by 40 cycles of: 95 °C for 15 s, and 60 °C for 1 min.

### Statistical methods

Statistical analyses were performed using the IBM SPSS Statistics 26 (SPSS Inc.). *χ*^2^ tests were used to compare categorical variables and the Mann–Whitney *U* test was used to compare differences in continuous variables between groups. Correlations between continuous variables were analysed using the Spearman´s rank correlation test. *P*-values < 0.05 were considered statistically significant. The area under the receiver operating characteristic (ROC) curve was calculated using the variable for *P. micra, F. nucleatum or clbA* + bacteria and cancer diagnosis/no cancer diagnosis. The Youden´s index was used to identify the cut-off for the different assays, resulting in an optimal trade-off between sensitivity and specificity in the detection of cancer. This cut-off was used to identify faecal samples as positive (with high levels of the indicated marker) or negative (with low levels of the indicated marker).

## Supplementary information

Supplementary information.

## Data Availability

The data that support the findings of this study are available from the corresponding author upon reasonable request.
