# Supplementary material for: Parvimonas micra as a putative non-invasive faecal biomarker for colorectal cancer
Source: Sci Rep. 2020 Sep 17;10:15250. doi: 10.1038/s41598-020-72132-1 (PMC7499209; doi:10.1038/s41598-020-72132-1)
Supplement: Supplementary file 1 — Supplementary information. [file 41598_2020_72132_MOESM1_ESM.pdf]

Supplementary Information

“*Parvimonas micra* as a putative non-invasive faecal biomarker for colorectal cancer”

Thyra Löwenmark[1], Anna Löfgren-Burström[1], Carl Zingmark[1], Vincy Eklöf[1], Michael Dahlberg[2], Sun Nyunt Wai[3], Pär Larsson[1], Ingrid Ljuslinder[4], Sofia Edin[1] and Richard Palmqvist[1]

[1]Department of Medical Biosciences, Pathology, Umeå University, Umeå, Sweden

[2]Department of Surgery, Sunderby Hospital, Luleå, Sweden

[3]Department of Molecular Biology, Umea University, Umeå, Sweden

[4]Department of Radiation Sciences, Oncology, Umeå University, Umeå, Sweden

Supplementary Table S1. Clinical characteristics of CRC patients from the FECSU cohort in relation to levels of *P. micra* in faeces.

	High n=23	Low n=15	*P*-value
**Age (%)**			
≤59	1 (4.3)	3 (20.0)	0.041
60-69	11(47.8)	1 (6.7)	
70-79	9 (39.1)	8 (53.3)	
≥80	2 (8.7)	3 (20.0)	
**Gender (%)**			
Female	11 (47.8)	7 (46.7)	0.944
Male	12 (52.2)	8 (53.3)	
**Location (%)**			0.967
Right colon	7 (30.4)	4 (26.7)	
Left colon	10 (43.5)	7 (46.7)	
Rectum	6 (26.1)	4 (26.7)	
**Stage (%)**			
I	1 (4.3)	1 (7.1)	0.936
II	12 (52.2)	8 (57.1)	
III	5 (21.7)	3 (21.4)	
IV	5 (21.7)	2 (14.3)	

Supplementary Table S2. Clinical characteristics of CRC patients from the U-CAN cohort in relation to levels of *P. micra* in faeces

	High<br>n= 135	Low<br>n= 103	*P*-value
**Age (%)**			
≤59	19 (14.1)	22 (21.4)	0.205
60-69	56 (41.5)	31 (30.1)	
70-79	42 (31.1)	38 (36.9)	
≥80	18 (13.3)	12 (11.7)	
**Gender (%)**			
Female	52 (38.5)	43 (41.7)	0.614
Male	83 (61.5)	60 (58.3)	
**Location (%)**			
Right colon	24 (17.8)	24 (23.3)	0.575
Left colon	24 (17.8)	17 (16.5)	
Rectum	87 (64.4)	62 (60.2)	
**Stage (%)**			
I	21 (16.5)	25 (25.5)	0.351
II	48 (37.8)	29 (29.6)	
III	37 (29.1)	28 (28.6)	
IV	21 (16.5)	16 (16.3)	

Supplementary Table S3. Primers and probes used for quantitative real-time PCR.

	**Forward**	**Reverse**	**Probe**	
P. micra	5'- AAGAATGGAGAGAG TTGTTAGAGAAAGAA - 3'	5'- TTGTGATAATTGTG AAGAACCGAAGA - 3'	5'- FAM- AACTCAAGATCCAGA CCTTGCTACGCCTCA - BHQ1 - 3'	1
F. nucleatum	5'- CAACCATTACTTTA ACTCTACCATGTTCA –3'	5'- GTTGACTTTACAGAAG GAGATTATGTAAAAATC -3'	5'- FAM- TCAGCAACTTGTCCTTCT TGATCTTTAAATGAACC - BHQ-1 -3'	2, 3
clbA	5'- ATGAGGATTGATA TATTAATTGGACA -3'	5'- GGTTTGCCATATTTGCA CGTAC -3'	SYBR Green I	4, 5
16S rRNA	5'- GGTGAATACGTTCC CGG - 3'	5'- TACGGCTACCTTGTTA CGACTT - 3'	SYBR Green I	2, 6, 7

1. Yu J, Feng Q, Wong SH, et al. Metagenomic analysis of faecal microbiome as a tool towards targeted non-invasive biomarkers for colorectal cancer. *Gut* 2017;66(1):70-78.
2. Flanagan L, Schmid J, Ebert M, et al. Fusobacterium nucleatum associates with stages of colorectal neoplasia development, colorectal cancer and disease outcome. *Eur J Clin Microbiol Infect Dis* 2014;33(8):1381-90.
3. Repass J, Reproducibility Project: Cancer B, Iorns E, et al. Replication Study: Fusobacterium nucleatum infection is prevalent in human colorectal carcinoma. *Elife* 2018;7.
4. Eklof V, Lofgren-Burstrom A, Zingmark C, et al. Cancer-associated fecal microbial markers in colorectal cancer detection. *Int J Cancer* 2017;141(12):2528-36.
5. Putze J, Hennequin C, Nougayrede JP, et al. Genetic structure and distribution of the colibactin genomic island among members of the family Enterobacteriaceae. *Infect Immun* 2009;77(11):4696-703.
6. Kostic AD, Chun E, Robertson L, et al. Fusobacterium nucleatum potentiates intestinal tumorigenesis and modulates the tumor-immune microenvironment. *Cell Host Microbe* 2013;14(2):207-15.
7. Xie YH, Gao QY, Cai GX, et al. Fecal Clostridium symbiosum for Noninvasive Detection of Early and Advanced Colorectal Cancer: Test and Validation Studies. *EBioMedicine* 2017;25:32-40.
